# Stability of within-host–parasite communities in a wild mammal system

**DOI:** 10.1098/rspb.2013.0598

**Published:** 2013-07-07

**Authors:** Sarah C. L. Knowles, Andy Fenton, Owen L. Petchey, Trevor R. Jones, Rebecca Barber, Amy B. Pedersen

**Affiliations:** 1 Institute of Evolutionary Biology, and Centre for Immunity, Infection and Evolution (CIIE), School of Biological Sciences, University of Edinburgh, West Mains Road, Edinburgh EH9 3JT, UK; 2 Department of Infectious Disease Epidemiology, Imperial College London, St. Mary's Campus, Norfolk Place, London W2 1PG, UK; 3 Institute of Integrative Biology, University of Liverpool, Biosciences Building, Crown Street, Liverpool L69 7ZB, UK; 4 Institute of Evolutionary Biology and Environmental Studies, University of Zurich, Winterthurerstrasse 190, 8057 Zurich, Switzerland; 5 Institute of Infection and Global Health, University of Liverpool, The Ronald Ross Building, 8 West Derby Street, Liverpool L69 7BE, UK

**Keywords:** co-infection, helminth, *Heligmosomoides polygyrus*, community ecology, interaction

## Abstract

Simultaneous infection by multiple parasite species is ubiquitous in nature. Interactions among co-infecting parasites may have important consequences for disease severity, transmission and community-level responses to perturbations. However, our current view of parasite interactions in nature comes primarily from observational studies, which may be unreliable at detecting interactions. We performed a perturbation experiment in wild mice, by using an anthelminthic to suppress nematodes, and monitored the consequences for other parasite species. Overall, these parasite communities were remarkably stable to perturbation. Only one non-target parasite species responded to deworming, and this response was temporary: we found strong, but short-lived, increases in the abundance of *Eimeria* protozoa, which share an infection site with the dominant nematode species, suggesting local, dynamic competition. These results, providing a rare and clear experimental demonstration of interactions between helminths and co-infecting parasites in wild vertebrates, constitute an important step towards understanding the wider consequences of similar drug treatments in humans and animals.

## 1. Introduction

Co-infection, where an individual host harbours multiple parasite species, is the rule rather than the exception in nature, and has been documented across diverse systems [1,2]. The parasites infecting an individual host can be viewed as an ecological community, within which species may interact directly through chemical or physical interference, indirectly via ‘bottom-up’ processes (e.g. competition for shared host resources) or indirectly via ‘top-down’ processes (e.g. immune-mediated competition or facilitation) [3].

Such interspecific interactions are an important determinant of how communities respond to external events. Specifically, the number, strength and arrangement of interactions among species will affect community stability in the face of perturbations (i.e. the removal or addition of organisms of a particular species) [4–6]. Many interactions, strong interactions and feedback loops all result in relatively unstable communities with both low resistance (perturbations have a large effect) and low resilience (long recovery times after perturbation) [4]. Furthermore, if the perturbed species is involved in many strong interactions with other species (i.e. it is a keystone species), then larger and more prolonged effects are expected than if the perturbed species has few or weak links to other species [7]. These and other determinants of stability are well researched in several types of ecological network, such as free-living food webs [8], but the stability of within-host–parasite communities to perturbation has yet to be assessed.

Understanding parasite interactions and the effects of perturbations is particularly important in disease ecology. A change in the abundance of a target parasite species, for example as a result of drug administration, could affect other (non-target) parasites in the same host, negatively or positively affecting host health depending on the type of parasite interaction. Thus, knowledge about the stability of within-host–parasite communities, and how interactions within them determine this, is highly valuable in both pure and applied disease research, and may be especially important for designing optimal treatment or vaccination strategies in co-infected populations.

Although within-host interactions between parasite species have frequently been demonstrated in laboratory experiments [9,10], these studies often use artificial infection regimes (e.g. single doses, often leading to infections of unnaturally high intensity), usually consider just two parasite species, and hosts are typically homogeneous (e.g. same sex and strain) and fed ad libitum, all of which will affect the relevance of these studies for natural host–parasite communities. Studies of wild hosts are therefore highly desirable. Conclusions from observational studies in wild populations have been mixed, with some suggesting within-host–parasite communities contain many strong interactions among species [11,12], whereas others conclude they are near-random assemblages with little structure and few interactions [13,14]. However, a well-known issue with observational approaches is that statistical associations between species may arise through a range of processes other than interspecific interaction [15]. For instance, for parasite species, this could involve covariance in exposure owing to host age or a shared transmission route. While one can attempt to control for confounding factors, the extent to which observed associations reflect true within-host–parasite interactions remains uncertain [16]. In free-living community ecology, manipulative (perturbation) field experiments are widely recognized as the most reliable way of measuring interspecific interactions and evaluating community stability [17]. For parasite communities, randomized, controlled treatment experiments constitute a powerful tool for measuring the strength of within-host–parasite interactions, assessing the timescale over which they operate, and directly measuring the stability of natural parasite communities to perturbations. However, they have rarely been adopted for examining within-host–parasite interactions (but see references [18,19]).

Here, we adopt a perturbation experimental approach to study natural within-host–parasite communities of wild wood mice (*Apodemus sylvaticus*). Our aims are twofold: (i) to determine the strength and nature of interactions among parasite species within these communities, and (ii) to assess the stability of these within-host communities to perturbation. We used the anthelminthic drug Ivermectin to reduce levels of nematode infection (the target parasite), and a longitudinal design to track the effects of this perturbation on both target and non-target parasites over the following weeks. Nematodes are an ideal target for such a perturbation experiment, as they are an abundant member of natural wood mouse parasite communities, and are well known for their immunomodulatory effects, in particular, their suppression of adaptive immune responses [20]. Consequently, nematodes may constitute keystone members of parasite communities, able to interact with many co-infecting parasites via the host's immune system [9,10,21] or by competition for resources in the gastrointestinal (GI) tract [22]. Thus, nematodes are expected to have many strong interactions with other parasites and, if parasite communities are relatively unstable, perturbing this group should have dramatic consequences for co-infecting parasite community members. Our experiment presents a first explicit test of these hypotheses.

## 2. Material and methods

### (a) Field experiment

Between May and December 2010, wood mice were trapped on five 0.5 ha grids in two woodlands in Cheshire (two grids in Manor Wood, 53°19′ N, 3°3′ W; three grids in Haddon Wood, 53°16′ N, 3°1′ W). An overview of the experimental design is given in the electronic supplementary material, figure S1. On each grid, two live traps (H. B. Sherman 2 × 2.5 × 6.5 inch folding trap, Tallahassee, FL, USA) baited with grain and bedding material were placed every 10 m in a 70 × 70 m square (total 128 traps per grid). Primary trapping was monthly: during the first three weeks of every month, two grids were trapped each week for three consecutive nights. At first capture, all mice were tagged using a subcutaneous microchip transponder for identification. On each grid, mice were rotationally assigned to one of three treatments at first capture: either repeated or single treatment with the anthelminthic Ivermectin, or a control treatment. Mice in the repeated Ivermectin treatment group received 10 mg kg^−1^ Ivermectin orally every primary trapping session (i.e. monthly, if captured every month). Those in the single-treatment group received this dose at first capture but water at subsequent captures. Control mice received water at every monthly capture. The two different treatment regimes were designed to test the impact of both a ‘press’ and a ‘pulse’ perturbation on parasite communities [17]. Ivermectin is a potent anti-nematode drug and is also known to affect some arthropods; however, we found no evidence that Ivermectin affected common ectoparasites on wood mice in this study (see electronic supplementary material, section S1). Ivermectin is not known to have direct effects on any other types of parasite. During the monthly *primary trapping* sessions, morphometric data were taken from all mice (age, sex, body mass, body length and reproductive condition), and a faecal and blood sample (from the tail tip) collected. Fur was also brushed to record tick and flea presence. Faecal samples were weighed and stored in 10 per cent formalin solution until parasite identification. During the last week of June, August and October, all experimental grids were trapped for an additional two consecutive nights each (*secondary trapping*, at which no treatments were given), permitting the collection of faeces from mice treated one to three weeks previously in a primary trapping session, and thus assessment of GI parasite responses to treatment over timescales shorter than a month (blood parasite data were available only for primary trapping sessions). GI parasites were detected using the salt flotation technique [22], and two blood parasite genera were screened for and identified using PCR-based diagnostics: bacteria of the genus *Bartonella* [23,24] and trypanosomes [25] (see the electronic supplementary material, section S2).

### (b) Statistical analysis of treatment effects

For parasites where infection was quantified by faecal egg counts (GI parasites such as nematodes and *Eimeria* spp.), two lines of reasoning motivated us to perform separate analyses of infection probability (parasite presence–absence) and infection intensity (log-transformed number of eggs/oocysts per gram faeces among infected individuals). First, the effects of treatment, and any revealed interspecific parasite interactions, could, in principle, arise from several biological processes affecting either (i) the probability of infection (e.g. through parasite establishment or clearance via drugs) and/or (ii) the success of parasites once inside the host, reflected either through parasite fecundity (for helminths), or replication ability (for microparasites such as *Eimeria* protozoa). Separate statistical analyses of infection probability and intensity allow us to tease apart these potential processes. Second, this analysis strategy was supported by results from zero-inflated negative binomial (ZINB) models [26] on egg/oocyst counts measured shortly after treatment (one to three weeks later; see §3). ZINB models allowed us to test whether treatment affected one or both of two statistical processes: a negative binomial count process, which accounts for the typically observed aggregated distribution in parasite egg/oocyst counts, and a zero-inflation process by which additional zero counts (i.e. individuals who are uninfected or who have been cleared of infection) are generated. By comparing the fit of a ZINB model with a standard negative binomial generalized linear model (GLM) for both nematodes and *Eimeria* spp., we tested whether the data suggested a need to consider effects on infection probability and parasite count separately, or whether a single negative binomial count process better represented the data. For both parasite groups, which had highly aggregated distributions with many zero counts (see the electronic supplementary material, figure S2), zero-inflated models provided a significantly better fit than the equivalent negative binomial models and suggested that, in the short-term, treatment had differential effects on a count and a zero-inflation process (see §3 and electronic supplementary material, section S3*a*). For blood microparasites (*Bartonella* bacteria and trypanosomes), where infection load could not be quantified, we tested treatment effects on infection probability using binomial GLMs, to assess whether treatment altered susceptibility to infection.

We analysed the effect of Ivermectin on target (nematodes) and non-target parasites in mice from the experimental grids over several timescales (one to three, four and eight weeks after first treatment), using GLMs in R v. 2.13 [27]. All models were performed with the glm function, with infection probability models using a binomial error distribution, whereas intensity models used a Gaussian error distribution. Parasite species from the same genera were pooled in the main analyses, but species with prevalence more than 10 per cent were also analysed individually where data permitted (table 1).

**Table 1.** The prevalence and infection site of gastrointestinal (GI) and blood parasites found in wood mouse samples taken during the parasite community perturbation experiment. Taxonomic groups of parasites whose prevalence exceeded 10% (shown in italics) were used as response variables in statistical analyses.

	site of infection	prevalence (%)	mean eggs/oocysts per gram faeces
macroparasites			
nematodes	GI tract	*46.78*	40 (range 0–673)
*Heligmosomoides polygyrus*	(upper ileum)	*42.03*	33 (range 0–563)
*Syphacia stroma*	(throughout tract)	5.42	
*Aonchotheca murissylvatici*		1.70	
Nematode sp. 1^a^		2.27	
*Aspiculuris* sp.		0.87	
Cestodes	GI tract	6.78	
*Hymenolepsis* sp.		3.05	
Cestode sp. 1^a^		1.02	
Cestode sp. 2^a^		3.05	
microparasites			
*Eimeria* spp.	GI tract	*46.10*	5448 (range 0–182 400)
*E. hungaryensis*	(Upper ileum)	*30.17*	2876 (range 0–181 000)
*E. apionodes*	(Lower ileum)	*14.92*	1625 (range 0–177 100)
*Eimeria* sp*.* 1^a^		9.83	
*E. uptoni*		2.03	
*Bartonella* spp.	vascular endothelium, followed by invasion of red blood cells	*58.07*	
*B. doshiae*-like		9.72	
*B. grahamii*		*18.98*	
*B. taylorii*		*31.94*	
*B. birtlesii*		7.87	
BGA		0.93	
*Trypanosoma grosi*	bloodstream (extracellular)	*12.09*	

^a^Parasites marked could not be identified to a lower taxinomic level based on egg/oocyst morphology alone.

In all models, treatment was represented either as a two-level factor (‘drug’: yes (including repeated and single treatments) or no) over timescales before treatments diverged (up to one month after first capture), or as a three-level factor (‘treatment’: single, repeated or control) for timescales where treatment groups differed (more than a month after first capture). For GI parasites, data from secondary trapping sessions were used to test for short-term effects of Ivermectin treatment (within three weeks of treatment), whereas longer-term treatment effects were tested at one and two months after first capture. For blood parasites, treatment effects were examined one and two months after first capture. Interaction terms between treatment and initial nematode status were examined, where initial nematode status was taken as infection status at first capture (for primary trapping data collected four and eight weeks after first capture), or infection status one to three weeks previously (for secondary trapping data). Relevant covariates were fitted in all models where appropriate: trapping grid (five-level factor), month of capture (factor), host age (three-level factor: juveniles, subadults and adults), host sex and faecal sample mass (continuous) or DNA concentration (continuous). Reproductive status was coded as a binary variable with animals deemed reproductively active if they had descended or protruding testes (males), were pregnant or had a perforate vagina (females).

We also tested whether treatment affected total within-host–parasite species richness (GI and blood parasites combined at monthly trapping sessions), or GI richness (at all trapping sessions), over the same timescales outlined earlier. In models of parasite richness, overdispersion was accounted for (if required) by using a quasi-Poisson model. All starting models were simplified by backward stepwise elimination of non-significant terms (*p* > 0.05) beginning with interactions, to obtain the minimum adequate model. We compared these results with those from an Akaike information criteria-based model selection approach [28] to confirm findings were robust to model selection method (electronic supplementary material, section S3*b*). Full details of covariates included in each starting model, and terms remaining in the minimal model after simplification, are given in electronic supplementary material, table S4.

### (c) Calculation of treatment effect sizes

An effect size (Hedge's *g*, the standardized mean difference) was calculated to measure the magnitude and direction of nematode treatment effect for each parasite species-specific response variable. This was performed on two timescales: (i) to measure immediate effects of treatment, one to three weeks after the last treatment was given (secondary trapping data), and (ii) four weeks after first capture. For *Eimeria* oocyst intensity (log-transformed), means and standard deviations in the control and treated groups were used to directly calculate *g* [29]. For infection probability, the odds ratio (OR) was first calculated, then standard conversion calculations [29] were used to derive Hedge's *g* from the OR, so effect sizes for parasite intensity and infection probability responses were on a common scale.

## 3. Results

The wood mice in these populations harboured a diverse community of parasites (table 1), with individuals being simultaneously infected with up to six parasite species at any one time (mean within-host–species richness = 2.09), and 66 per cent of individuals co-infected. Overall, 146 mice in the experiment were captured a total of 312 times, with a mean number of captures per mouse of 2.14 (range 1–8). Few initial differences between treatment groups in parasite response variables were present, though these were found for *Eimeria hungaryensis* and pooled *Bartonella* spp. infection probability, as well as *Eimeria apionodes* intensity (see the electronic supplementary material, table S1).

### (a) Effect of anti-nematode treatment on target parasites

**Figure 1.**
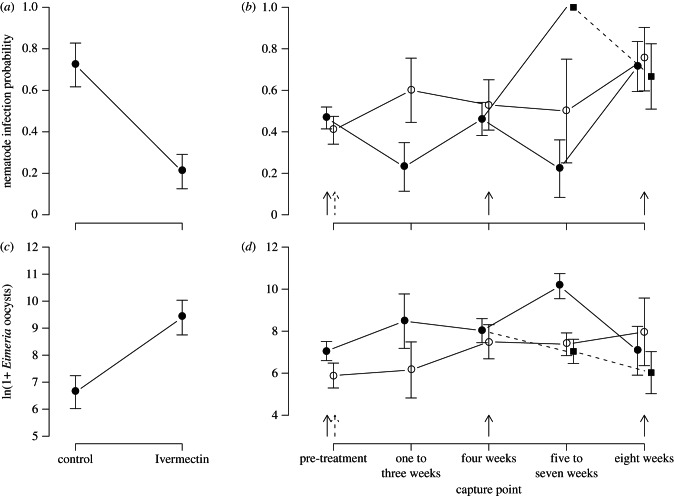
Effects of the anthelminthic drug Ivermectin on target (nematodes) and non-target (*Eimeria*) parasites. (*a*) Effect of Ivermectin administered one to three weeks previously on nematode infection probability. (*b*) Nematode infection probability over time since first capture (*c*) Effect of Ivermectin administered one to three weeks previously on *Eimeria* spp. infection intensity. (*d*) *Eimeria* spp. infection intensity over time since first capture. In (*b*) and (*d*), arrows indicate times of treatment (solid arrows denote repeated treatment; dashed arrows denote single treatment); single-treatment mice (dashed line, filled squares) are grouped with repeated treatment mice (solid line, filled circles) until four weeks, at which point they become different. Data are means and s.e.m. from raw data.

Anthelminthic treatment had a clear negative effect on nematodes (the target parasites; figure 1*a*). A ZINB model of nematode egg counts one to three weeks after treatment provided a better fit to the data than a standard negative binomial model (Vuong test, *p* = 0.008). This result suggests that shortly after treatment, egg counts were better represented by the combination of a count process and an additional zero-generating process (i.e. individuals clearing their nematode infection), rather than a count process alone. This ZINB model revealed a strong effect of Ivermectin on the probability of being uninfected post-treatment (OR = 10.03 of a zero count for treated compared with untreated mice; normal 95% CI: 2.30, 43.77), but weaker evidence that treatment reduced egg count (incidence rate ratio, IRR = 0.379 for treated compared with untreated mice, normal 95% CI: 0.121, 1.183; electronic supplementary material, section S3*a*). In line with the ZINB model results, a binomial model of infection probability (which controlled for more covariates as shown in electronic supplementary material, table S2) showed that treated mice had a 71 per cent lower probability of infection three weeks after treatment compared with control mice (figure 1*a*; electronic supplementary material, table S2; drug: *χ*^2^_1_ = 15.52, *p* < 0.001, *n* = 42). Post hoc tests revealed no evidence that the treatment effect of reduced infection probability varied between months, across host age groups (adult versus subadult), with body mass or with reproductive status (drug × month *χ*^2^_1_ = 0.50, *p* = 0.479; drug × age *χ*^2^_1_ = 1.53, *p* = 0.216; drug × body mass *χ*^2^_1_ = 0.027, *p* = 0.870, drug × reproductive status *χ*^2^_1_ = 2.52, *p* = 0.112). No differences in infection probability were detectable one or two months after first treatment (one month: drug *χ*^2^_1_ = 2.33, *p* = 0.127, *n* = 56; two months: treatment *χ*^2^_1_ = 0.75, *p* = 0.688, *n* = 31), indicating that the effect of Ivermectin on nematodes was short-lived. Finally, as the results above indicate, not all treated mice were completely cleared of nematodes (five of 24 treated mice had nematode eggs in faeces one to three weeks after Ivermectin administration). The following analyses include these individuals, however, their exclusion made very little quantitative difference to analyses, and no difference to our conclusions.

The temporal dynamics of nematode infection probability also clearly reflected the pattern of Ivermectin treatment (figure 1*b*). Notably, mice in the repeated treatment group showed a reduction in nematode infection probability after each treatment, whereas those in the single-treatment group exhibited a treatment effect only immediately after their treatment (figure 1*b*; single versus repeated nematode infection probability five to seven weeks after first capture, *χ*^2^_1_ = 9.87, *p* = 0.0017). The most common nematode (*Heligmosomoides polygyrus*, accounting for 90% of nematode-infected mice, 42% prevalence overall; table 1) exhibited qualitatively similar treatment effects (electronic supplementary material, figure S3), suggesting this species drives the effects on overall nematode infection probability. Ivermectin had no detectable effect on nematode infection intensity (egg counts) among infected individuals over any timescale considered (one to three weeks post-treatment: *F*_1,13_ = 1.17, *p* = 0.299; one month after first capture: drug *F*_1,12_ = 0.00, *p* = 0.978; two months after first capture: treatment *F*_2,19_ = 1.81, *p* = 0.190; electronic supplementary material, figure S4).

### (b) Effects of anti-nematode treatment on non-target parasites

As with nematodes, a ZINB model on *Eimeria* oocyst counts one to three weeks after treatment provided a better fit than a standard negative binomial model (Vuong test, *p* = 0.003). This model indicated that treatment increased *Eimeria* oocyst counts (IRR = 16.45 for treated compared with untreated mice, normal 95% CI: 5.02, 53.84), but did not significantly influence the probability of *Eimeria* infection (OR = 2.12, normal 95% CI: 0.61, 7.43; electronic supplementary material, section S3*a*). *Eimeria* oocyst counts were therefore over 15 times higher in Ivermectin-treated mice compared with untreated mice one to three weeks after treatment (GLM on logged oocyst counts, *F*_1,15_ = 8.93, *p* = 0.009; figure 1*c* and electronic supplementary material, table S2). Post hoc tests showed this effect did not vary significantly between months, or with host age (adult versus subadult) or reproductive status (drug × month *F*_1,14_ = 0.00, *p* = 0.986; drug × age *F*_1,13_ = 0.37, *p* = 0.554; drug × reproductive status *F*_1,13_ = 0.02, *p* = 0.901).

*Eimeria* parasites also responded rapidly to nematode reinfection post-treatment, with *Eimeria* infection intensity declining over the same timescale that nematode infection probability increased: thus, the dynamics of *Eimeria* spp. intensity mirrored those of nematode infection probability, showing temporary increases after each Ivermectin treatment, rapidly returning to pre-perturbation levels as nematodes re-infected (figure 1*d*). Just as for nematodes, no treatment differences in *Eimeria* intensity were detectable one or two months after first capture (one month: drug *F*_1,23_ = 0.10, *p* = 0.760; two months: treatment *F*_2,10_ = 1.17, *p* = 0.348), indicating treatment-induced increases in *Eimeria* intensity were similarly short-lived (figure 1*d*). As with nematode infection probability, the repeated and single-treatment groups diverged in their *Eimeria* intensity dynamics beyond one month after first capture (figure 1*d*; single versus repeated *Eimeria* infection intensity five to seven weeks after first capture, *χ*^2^_1_ = 7.11 *p* = 0.008), which further supports the conclusion that these *Eimeria* intensity dynamics were driven by nematode treatment.

*Eimeria* spp. infection probability was not significantly altered by treatment over any timescale examined, whether considering *Eimeria* species pooled or separately (electronic supplementary material, figure S5; pooled *Eimeria* spp.: within three weeks of last treatment, drug: *χ*^2^_1_ = 0.76, *p* = 0.383, *n* = 42; one month after first capture: drug *χ*^2^_1_ = 1.24, *p* = 0.265, *n* = 56; two months after first capture: treatment *χ*^2^_2_ = 1.84, *p* = 0.399, *n* = 31; *p* > 0.10 for all drug or treatment terms in equivalent models for the individual species *E. hungaryensis* and *E. apionodes*).

**Figure 2.**
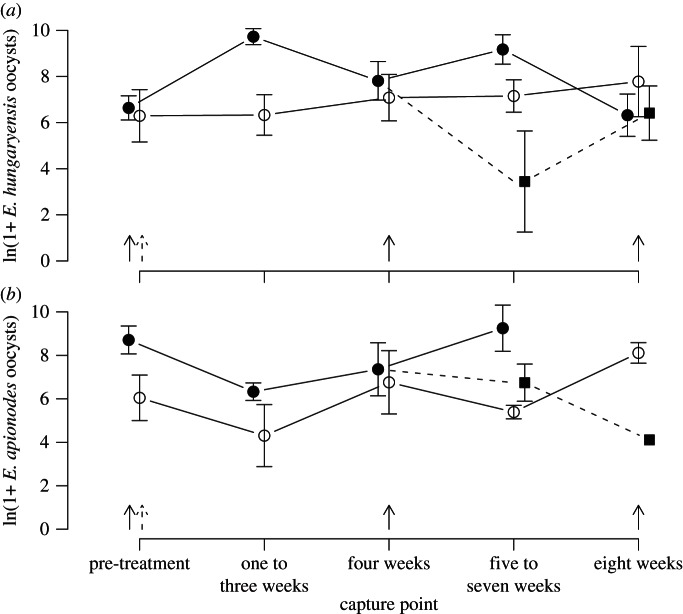
Infection intensity over time since first capture for (*a*) *Eimeria hungaryensis* and (*b*) *Eimeria apionodes* across Ivermectin treatment groups. While *E. hungaryensis* infection intensity increased after each treatment, and more closely mirrored the dynamics of nematode infection probability, *E. apionodes* was less affected by treatment. Arrows indicate times of treatment (solid arrows denote repeated treatment; dashed arrows denote single treatment); single-treatment mice (dashed line, filled squares) are grouped with repeated treatment mice (solid line, filled circles) until four weeks, at which point they become different. Data are means and s.e.m. from raw data.

Examining the *Eimeria* intensity response in more detail, we found evidence for species-specificity in this effect. Two species of *Eimeria,* differing in their infection site within the gut, are common in these wood mouse populations: *E. hungaryensis* and *E. apionodes. Eimeria hungaryensis* is found in the anterior half of the small intestine (where *H. polygyrus*, the most common nematode in these mice, resides; table 1) and predominantly infects enterocytes on the apex of villi, whereas *E. apionodes* inhabits a more posterior position in the gut, infecting enterocytes on the sides of villi or in crypts [30]*.* Ivermectin-treated mice showed a stronger increase in *E. hungaryensis* infection intensity than in *E. apionodes* intensity (one to three weeks after last treatment, effect of drug for *E. hungaryensis*: *F*_1,12_ = 13.66, *p* = 0.003, *E. apionodes*: *F*_1,11_ = 6.74, *p* = 0.025), and the dynamics of *E. hungaryensis* intensity mirrored those of nematode infection probability far more closely than the dynamics of *E. apionodes* (compare figure 2 with figure 1*b*).

No treatment effects on the probability of infection with blood parasites (either pooled or individual *Bartonella* spp. or *Trypanosoma grosi*) were detected one or two months after treatment (see the electronic supplementary material, figure S6; pooled *Bartonella* spp. one month: drug *χ*^2^_1_ = 0.02, *p* = 0.878, *n* = 47; two months: treatment *χ*^2^_2_ = 0.24, *p* = 0.885, *n* = 24. *Trypanosoma* one month: *χ*^2^_1_ = 0.19, *p* = 0.662, *n* = 46; two months: *χ*^2^_2_ = 3.76, *p* = 0.153, *n* = 24; *p* > 0.35 for drug or treatment effects in equivalent models for the individual species *Bartonella taylorii* and *Bartonella grahamii*). Similarly, we found no effect of nematode treatment on total GI parasite richness, either including or excluding nematodes, in the subsequent three weeks (including nematodes: drug *χ*^2^_1_ = 1.83, *p* = 0.176, *n* = 42; excluding nematodes: drug *χ*^2^_1_ = 0.17, *p* = 0.679, *n* = 42).

**Figure 3.**
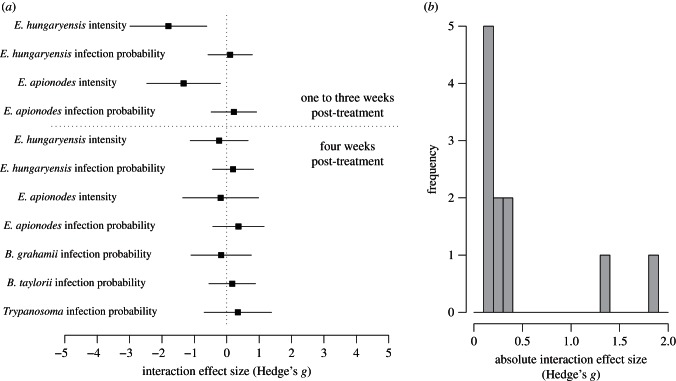
(*a*) Summary of Ivermectin treatment effects on non-target parasites. Effect size estimates (Hedge's *g*) for the effect of treatment on non-target parasite response variables. Negative *g*-values indicate suppressive effects of nematodes (treatment resulted in higher infection intensities or infection probability for the non-target parasite than control mice), positive values indicate the opposite. Effect sizes were calculated either for parasite infection probability (using the odds ratio to calculate *g*), or from the difference in mean oocyst counts per gram faeces, on a log scale as used in statistical analyses. (*b*) Frequency histogram of the absolute effect sizes shown in (*a*), showing skew of the distribution towards primarily weak interactions.

There were also no differences in total parasite richness or GI richness (including or excluding nematodes) at one or two months after first capture (*p* > 0.25, for drug or treatment terms in all cases) and overall, parasite community richness was not notably altered by Ivermectin treatment over any timescale (see the electronic supplementary material, figure S7). Considering all parasite responses examined, non-target treatment effects were predominantly weak, with only a few strong, negative interactions detected (figure 3*a*), and the distribution of absolute treatment effect sizes for non-target parasite species was highly skewed towards zero (figure 3*b*).

## 4. Discussion

Using a novel drug-based perturbation experimental approach, we provide a rare experimental demonstration of interaction between co-infecting parasite species in a wild vertebrate population, but also show that, overall, the within-host–parasite communities examined were relatively stable to drug-based perturbation. Mice given Ivermectin showed a reduction in nematode infection probability, while concurrently showing more than a 15-fold increase in *Eimeria* intensity compared with controls, suggesting significant competition between these two GI parasite taxa. Moreover, this interaction appeared to be highly dynamic, with *Eimeria* intensity rapidly returning to pre-perturbation levels as nematode re-infection occurred (figure 1).

Although we cannot categorically exclude the possibility that these effects are caused by a direct positive effect of the anti-nematode drug on *Eimeria* parasites, rather than an indirect effect caused by a reduction in nematodes, a direct effect seems unlikely for two reasons. First, although macrocyclic lactones, including Ivermectin, are known to have non-specific effects on other organisms besides nematodes, including arthropods, protozoa and bacteria [31–33], these tend to be negative, and we can find no instances of direct positive effects on non-target parasites in the literature, despite widespread use of Ivermectin in livestock, which are frequently co-infected. Second, we find evidence for species-specificity in the effect on *Eimeria*, which seems more plausibly attributed to nematode removal than a species-specific direct effect of Ivermectin. Ivermectin treatment had a much clearer positive effect on *E. hungaryensis*, which shares an infection site with the dominant nematode in this system (*H. polygyrus*; table 1), than *E. apionodes*, which occupies a lower section of the GI tract (figure 2). These findings strongly suggest the effects of Ivermectin on *Eimeria* are due to the drug's negative effect on *H. polygyrus*, and reveal an extremely localized competitive interaction occurring between a pair of species that inhabit the same section of the GI tract. Interestingly, a controlled co-infection experiment in laboratory mice involving *H. polygyrus* and *Eimeria vermiformis*, a species that inhabits the caecum, did not find competitive effects as we report here and, if anything, showed some facilitation of *Eimeria* by early, but not chronic stage *H. polygyrus* infection [34]. This suggests that, as with free-living systems, species-specific ecological differences are likely important determinants of interaction mechanism, direction and strength among parasites.

The mechanism underlying the localized competition we identified is unknown. However, the fact that we detected an effect of anti-nematode treatment on *Eimeria* intensity, but not on the probability of being *Eimeria* infected, suggests that whereas nematodes do not alter susceptibility to acquiring *Eimeria* infection per se, they do affect the extent to which *Eimeria* parasites replicate once inside the GI tract. We suggest several potential, non-mutually exclusive mechanisms by which this could occur. First, it could arise by a resource-mediated interaction, with *H. polygyrus* and *Eimeria* parasites competing for space or nutrients with the gut. Second, because *H. polygyrus* feeds on epithelial tissue [35], in which *Eimeria* parasites develop, direct predation of *Eimeria* parasites by *H. polygyrus* may take place. Third, immune mechanisms may be involved, for example, an immune-mediated increase in enterocyte turnover during nematode infection, that could aid the host in *Eimeria* expulsion [36].

Although nematodes are expected to be a key interactive group because of their immunomodulatory effects [9,20,21], in this experiment the majority of parasites, as well as community richness as a whole, showed no detectable response to nematode suppression, producing a distribution of interaction strengths skewed towards zero. Moreover, non-target parasites that did respond (*Eimeria* spp.) quickly returned to pre-perturbation levels. Hence, these naturally occurring parasite communities appear to be both resistant and resilient to nematode-targeted perturbation. Theory suggests several mechanisms could underpin such community stability. First, the target parasite group (nematodes, and *H. polygyrus* particularly) may have few or relatively weak interactions with co-infecting parasites, although this would run counter to expectations based on many laboratory studies [9,10,37]. A second possibility is that shifts in community structure caused by nematode treatment may be extremely short-lived, and simply not visible over the timescales monitored (weeks to months). The one non-target response we found, involving *Eimeria* spp., was detectable within weeks, but not months of treatment, suggesting these parasite communities are extremely resilient and can recover quickly (within four weeks) from perturbations. A number of studies on the response of human gut microbial communities to antibiotic perturbation have documented similar resilience, whereby communities show notable shifts in the weeks following treatment but largely rebound to their original state over months or years [38,39]. Third, it may be that the structure of within-host–parasite communities themselves is conducive to stability. In particular, the skewed distribution of interaction strengths observed here (figure 3*b*) echoes findings from food webs of free-living species [40,41], and is known to promote network stability [7]. Furthermore, stability can also arise through high modularity or compartmentalization, where a network comprises modules of strongly interacting species, with weak or few links between modules [6]. It is noteworthy in this respect that the only significant interaction detected here was between two parasites that live in close proximity to each other. Interestingly, a recent literature-based network study of human co-infection data found significant modularity in the parasite community, with distinct modules of closely interacting species largely reflecting different locations in the body (E. C. Griffiths, A. B. Pedersen, A. Fenton & O. L. Petchey 2012, unpublished data). Thus, it may be that although strong interactions between co-infecting parasites do occur within any given module (e.g. body compartment), links to other such groups (e.g. through systemic immune effects) are rare or weaker, facilitating overall stability of the network in the face of perturbation.

The drug-based perturbations we used resemble current anthelmintic treatment programmes in humans and livestock, in terms of the drug used (Ivermectin), the periodic nature of treatment [42], efficacy level [43] and observed responses post-treatment, with rapid helminth reinfection after drug administration [44,45]. Our study shows, for the first time in a natural mammalian host–parasite system, how such treatments may impact the wider parasite community, leading to unintended responses in the abundance of some co-infecting parasites, but not in others. We also show how a community ecology framework can help identify how the strength, type and arrangement of parasite interactions may contribute to within-host–parasite community stability, and pinpoint certain parasite characteristics (e.g. shared location within the host) that predispose non-target parasites to respond to treatment. Such perturbation studies on natural parasite communities provide direct empirical demonstration of how anthelminthic treatment may impact the wider parasite community, and can inform our understanding of whether ‘holistic’ disease control programmes, that seek an indirect, added benefit of deworming through parasite interactions [46,47] are likely to achieve their goal.

## References

[1] Petney T Andrews R 1998 Multiparasite communities in animals and humans: frequency, structure and pathogenic significance. Int. J. Parasitol. 28, 377–393 10.1016/S0020-7519(97)00189-6 (doi:10.1016/S0020-7519(97)00189-6) 9559357

[2] Rigaud T Perrot-Minnot M-J Brown MJF 2010 Parasite and host assemblages: embracing the reality will improve our knowledge of parasite transmission and virulence. P. R. Soc. B 277, 3693–3702 10.1098/rspb.2010.1163 (doi:10.1098/rspb.2010.1163) PMC2992712 20667874

[3] Pedersen AB Fenton A 2007 Emphasizing the ecology in parasite community ecology. Trends Ecol. Evol. 22, 133–139 10.1016/j.tree.2006.11.005 (doi:10.1016/j.tree.2006.11.005) 17137676

[4] Pimm S 1984 The complexity and stability of ecosystems. Nature 307, 321–326 10.1038/307321a0 (doi:10.1038/307321a0)

[5] Neutel A-M Heesterbeek JAP de Ruiter PC 2002 Stability in real food webs: weak links in long loops. Science 296, 1120–1123 10.1126/science.1068326 (doi:10.1126/science.1068326) 12004131

[6] Stouffer DB Bascompte J 2011 Compartmentalization increases food-web persistence. Proc. Natl Acad. Sci. USA 108, 3648–3652 10.1073/pnas.1014353108 (doi:10.1073/pnas.1014353108) 21307311 PMC3048152

[7] Solé RV Montoya M 2001 Complexity and fragility in ecological networks. Proc. R. Soc. Lond. B 268, 2039–2045 10.1098/rspb.2001.1767 (doi:10.1098/rspb.2001.1767) PMC1088846 11571051

[8] Ings TC 2009 Ecological networks: beyond food webs. J. Anim. Ecol. 78, 253–269 10.1111/j.1365-2656.2008.01460.x (doi:10.1111/j.1365-2656.2008.01460.x) 19120606

[9] Graham AL 2008 Ecological rules governing helminth-microparasite coinfection. Proc. Natl Acad. Sci. USA 105, 566–570 10.1073/pnas.0707221105 (doi:10.1073/pnas.0707221105) 18182496 PMC2206576

[10] Knowles SCL 2011 The effect of helminth co-infection on malaria in mice: a meta-analysis. Int. J. Parasitol. 41, 1041–1051 10.1016/j.ijpara.2011.05.009 (doi:10.1016/j.ijpara.2011.05.009) 21777589

[11] Lello J Boag B Fenton A Stevenson I Hudson P 2004 Competition and mutualism among the gut helminths of a mammalian host. Nature 428, 840–844 10.1038/nature02490 (doi:10.1038/nature02490) 15103373

[12] Telfer S Lambin X Birtles R Beldomenico P Burthe S Paterson S Begon M 2010 Species interactions in a parasite community drive infection risk in a wildlife population. Science 330, 243–246 10.1126/science.1190333 (doi:10.1126/science.1190333) 20929776 PMC3033556

[13] Poulin R 1996 Richness, nestedness, and randomness in parasite infracommunity structure. Oecologia 105, 545–551 10.1007/BF00330018 (doi:10.1007/BF00330018) 28307148

[14] Behnke JM 2008 Structure in parasite component communities in wild rodents: predictability, stability, associations and interactions…or pure randomness? Parasitology 135, 1–16 10.1017/S0031182008000334 (doi:10.1017/S0031182008000334) 18371244

[15] Schluter D 1984 A variance test for detecting species associations, with some example applications. Ecology 65, 998–1005 10.2307/1938071 (doi:10.2307/1938071)

[16] Fenton A Viney ME Lello J 2010 Detecting interspecific macroparasite interactions from ecological data: patterns and process. Ecol. Lett. 13, 606–615 10.1111/j.1461-0248.2010.01458.x (doi:10.1111/j.1461-0248.2010.01458.x) 20529102

[17] Bender E Case T Gilpin M 1984 Perturbation experiments in community ecology: theory and practice. Ecology 65, 1–13 10.2307/1939452 (doi:10.2307/1939452)

[18] Ferrari N Cattadori I Rizzoli A Hudson P 2009 *Heligmosomoides polygyrus* reduces infestation of *Ixodes ricinus* in free-living yellow-necked mice, *Apodemus flavicollis*. Parasitology 136, 305–316 10.1017/S0031182008005404 (doi:10.1017/S0031182008005404) 19154651

[19] Pedersen AB Antonovics A 2013 Nematode treatment alters the parasite community in a wild mouse host. Biol. Lett. 10.1098/rsbl.2013.0205 PMC3730629 23658004

[20] Maizels R Balic A Gomez-Escobar N Nair M Taylor MD Allen JE 2004 Helminth parasites: masters of regulation. Immunol. Rev. 201, 89–116 10.1111/j.0105-2896.2004.00191.x (doi:10.1111/j.0105-2896.2004.00191.x) 15361235

[21] van Riet E Hartgers FC Yazdanbakhsh M 2007 Chronic helminth infections induce immunomodulation: consequences and mechanisms. Immunobiology 212, 475–490 10.1016/j.imbio.2007.03.009 (doi:10.1016/j.imbio.2007.03.009) 17544832

[22] Holmes J 1961 Effects of concurrent infections on *Hymenolepsis diminuta* (Cestoda) and *Monoliformis dubius* (Acanthocephala). I. General effects and comparison with crowding. J. Parasitol. 47, 209–216 10.2307/3275291 (doi:10.2307/3275291) 13715464

[23] Pritchard M Kruse G 1982 The collection and preservation of animal parasites. Lincoln, NE: University of Nebraska Press

[24] Telfer S Bown KJ Sekules R Begon M Hayden T Birtles R 2005 Disruption of a host–parasite system following the introduction of an exotic host species. Parasitology 130, 661–668 10.1017/S0031182005007250 (doi:10.1017/S0031182005007250) 15977903

[25] Noyes H Stevens J Teixeira M Phelan J Holz P 1999 A nested PCR for the ssrRNA gene detects *Trypanosoma binneyi* in the platypus and *Trypanosoma* sp. in wombats and kangaroos in Australia. Int. J. Parasitol. 29, 331–339 10.1016/S0020-7519(98)00167-2 (doi:10.1016/S0020-7519(98)00167-2) 10221634

[26] Hilbe JM 2011 Negative binomial regression, 2nd edn New York, NY: Cambridge University Press

[27] R Development Core Team 2011 R: a language and environment for statistical computing. Vienna, Austria: R Foundation for Statistical Computing

[28] Bartoń K 2011 *MuMIn: Multi-model inference* (R package version 1.0.0 Vienna, Austria: R Foundation for Statistical Computing See http://CRAN.R-project.org/package=MuMIn

[29] Cooper H Hedges LV 2009 The handbook of research synthesis and meta-analysis, 2nd edn New York, NY: Russell Sage Foundation

[30] Nowell F Higgs S 1989 *Eimeria* species infecting wood mice (genus *Apodemus*) and the transfer of two species to *Mus musculus*. Parasitology 98, 329–336 10.1017/S0031182000061394 (doi:10.1017/S0031182000061394) 2528109

[31] Arafa MA Wanas MQ 1996 The efficacy of ivermectin in treating rabbits experimentally infected with *Eimeria* as indicated parasitologically and histologically. J. Egypt Soc. Parasitol. 26, 773–780 8918049

[32] Lumaret J-P Errouissi F Floate K Römbke J Wardhaugh K 2012 A review on the toxicity and non-target effects of macrocyclic lactones in terrestrial and aquatic environments. Curr. Pharm. Biotechnol. 13, 1004–1060 10.2174/138920112800399257 (doi:10.2174/138920112800399257) 22039795 PMC3409360

[33] Lim LE Vilchèze C Ng C Jacobs WR Jr Ramón-García S Thompson CJ 2013 Anthelmintic avermectins kill *Mycobacterium tuberculosis*, including multidrug-resistant clinical strains. Antimicrob. Agents Chemother. 57, 1040–1046 10.1128/AAC.01696-12 (doi:10.1128/AAC.01696-12) 23165468 PMC3553693

[34] Rausch S Held J Stange J Lendner M Hepworth MR Klotz C Lucius R Pogonka T Hartmann S 2010 A matter of timing: early, not chronic phase intestinal nematode infection restrains control of a concurrent enteric protozoan infection. Eur. J. Immunol. 40, 2804–2815 10.1002/eji.201040306 (doi:10.1002/eji.201040306) 20809519

[35] Bansemir A Sukhdeo M 1994 The food resource of adult *Heligmosomoides polygyrus* in the small intestine. J. Parasitol. 80, 24–28 10.2307/3283340 (doi:10.2307/3283340) 8308654

[36] Artis D Grencis RK 2008 The intestinal epithelium: sensors to effectors in nematode infection. Mucosal Immunol. 1, 252–264 10.1038/mi.2008.21 (doi:10.1038/mi.2008.21) 19079187

[37] Ezenwa VO Jolles AE 2011 From host immunity to pathogen invasion: the effects of helminth coinfection on the dynamics of microparasites. Integr. Comput. Biol. 51, 540–551 10.1093/icb/icr058 (doi:10.1093/icb/icr058) 21727178

[38] Jernberg C Lofmark S Edlund C Jansson JK 2010 Long-term impacts of antibiotic exposure on the human intestinal microbiota. Microbiology 156, 3216–3223 10.1099/mic.0.040618-0 (doi:10.1099/mic.0.040618-0) 20705661

[39] Dethlefsen L Relman DA 2011 Incomplete recovery and individualized responses of the human distal gut microbiota to repeated antibiotic perturbation. Proc. Natl Acad. Sci. USA 108, 4554–4561 10.1073/pnas.1000087107 (doi:10.1073/pnas.1000087107) 20847294 PMC3063582

[40] Berlow EL Navarrete S Briggs C Power M Menge B 1999 Quantifying variation in the strengths of species interactions. Ecology 80, 2206–2224 10.1890/0012-9658(1999)080[2206:QVITSO]2.0.CO;2 (doi:10.1890/0012-9658(1999)080[2206:QVITSO]2.0.CO;2)

[41] Sala E Graham MH 2002 Community-wide distribution of predator-prey interaction strength in kelp forests. Proc. Natl Acad. Sci. USA 99, 3678–3683 10.1073/pnas.052028499 (doi:10.1073/pnas.052028499) 11891292 PMC122583

[42] Hotez P 2011 Enlarging the ‘audacious goal’: elimination of the world's high prevalence neglected tropical diseases. Vaccine 29(Suppl. 4), D104–D110 10.1016/j.vaccine.2011.06.024 (doi:10.1016/j.vaccine.2011.06.024) 22188933

[43] Geary TG 2010 Unresolved issues in anthelminthic pharmacology for helminthiases of humans. Int. J. Parasitol. 40, 1–13 10.1016/j.ijpara.2009.11.001 (doi:10.1016/j.ijpara.2009.11.001) 19932111

[44] Bundy DA Thompson DE Cooper ES Golden MH Anderson RM 1985 Population dynamics and chemotherapeutic control of *Trichuris trichiura* infection of children in Jamaica and St. Lucia. Trans. R. Soc. Trop. Med. H 79, 759–774 10.1016/0035-9203(85)90110-5 (doi:10.1016/0035-9203(85)90110-5) 3832488

[45] Njongmeta L Nfon CK Gilbert J Makepeace BL Tanya VN Trees AJ 2004 Cattle protected from onchocerciasis by ivermectin are highly susceptible to infection after drug withdrawal. Int. J. Parasitol. 34, 1069–1074 10.1016/j.ijpara.2004.04.011 (doi:10.1016/j.ijpara.2004.04.011) 15313133

[46] Bentwich Z Kalinkovich A Weisman Z Borkow G Beyers N Beyers AD 1999 Can eradication of helminthic infections change the face of AIDS and tuberculosis? Immunol. Today 20, 485–487 10.1016/S0167-5699(99)01499-1 (doi:10.1016/S0167-5699(99)01499-1) 10529774

[47] Druilhe P Tall A Sokhna C 2005 Worms can worsen malaria: towards a new means to roll back malaria? Trends Parasitol. 21, 359–362 10.1016/j.pt.2005.06.011 (doi:10.1016/j.pt.2005.06.011) 15967721

